# Black-tie dress code: two new species of the genus *Toxomerus* (Diptera, Syrphidae)

**DOI:** 10.3897/zookeys.140.1930

**Published:** 2011-10-26

**Authors:** Ximo Mengual

**Affiliations:** 1 Department of Entomology, NMNH, Smithsonian Institution, Washington, D.C. U.S.A.

**Keywords:** *Toxomerus*, flower flies, Syrphidae, new species, identification key

## Abstract

*Toxomerus hauseri* Mengual **sp. n.** and *Toxomerus picudus* Mengual **sp. n.** are described from Peru and Ecuador respectively. *Toxomerus circumcintus* (Enderlein, 1938) is treated as a valid species and not considered synonym of *Toxomerus marginatus*, and *Toxomerus ovatus* (Hull, 1942) is considered junior synonym of *Toxomerus nitidus* (Schiner, 1868). An identification key for the *Toxomerus* species with dark abdomens is given along with diagnoses for each studied species.

## Introduction

The tribe Toxomerini (Diptera: Syrphidae) comprises the single genus *Toxomerus* Macquart, 1855. Toxomerini is endemic to the New World, from southern Canada to southern Argentina and Chile (Thompson and Thompson 2006). There are only 6 endemic Nearctic species and more than 130 Neotropical species of *Toxomerus* (Borges and Couri 2009; Thompson et al. 2010), although only 101 species are published and validly named (Thompson 2010). *Toxomerus* species are one of the most abundant flower flies in the New World and they are typically relatively small, usually about 6 mm. Nevertheless, there are some species larger than 9 mm (see Metz and Thompson 2001 for a review). Adults feed on pollen and nectar acting as flower pollinators (Thompson and Thompson 2006; Ssymank and Kearns 2009), but data about larval feeding habits of *Toxomerus* are limited. Most of the known species larvae are predacious feeding frequently on soft-bodied Hemiptera, but also on Acari, Thysanoptera and larvae of Lepidoptera (Rojo et al. 2003). However, there are two well-known pollen-feeder species, *Toxomerus politus* (Say, 1823) (Riley and Howard 1888; Marín A. 1969) and *Toxomerus apegiensis* (Harbach, 1974) (Reemer and Rotheray 2009).

Enderlein (1938) established the tribe Toxomerini for *Toxomerus* and eight other genera but it was Vockeroth (1969) who recognized and re-classified this tribe as monogeneric. Toxomerini taxonomy is based mostly on the characteristic markings of the abdominal tergites and the male genitalia. Unfortunately, the abdominal pattern of some species may show a great variation and it may appear obscured or lost (Curran 1930; Hull 1943; Thompson 1981). Hull (1943) provided the last key for the genus but he did not include all the species. Metz and Thompson (2001) provided an excellent overview on the systematics of *Toxomerus*. More recently, Borges and Couri (2009) presented a key for the Brazilian species of *Toxomerus* with very helpful illustrations.

The monophyly of *Toxomerus* is supported by morphological characters (Vockeroth 1969, 1992; Thompson 1981) and molecular evidence (Mengual et al. 2008) but there is no subgeneric classification for *Toxomerus* (Hull 1943; Vockeroth 1969; Metz and Thompson 2001). Hull (1943) indicated in a scheme the possible relationships among *Toxomerus* species groups based on the abdominal pattern. In that diagram, Hull grouped together the species with uniformly black abdomen or abdomen reddish and gave two examples, *Toxomerus anthrax* (Schiner, 1868) and *Toxomerus nitidus* (Schiner, 1868).

The aim of this study is to describe two new, very distinct *Toxomerus* species with uniformly black abdomen and to provide an identification key and some diagnostic notes for *Toxomerus* species without a clear yellow/black abdominal pattern. I do not think that “black abdomen” species are a natural group but are a phenotypic cluster instead. However, the abdomen without medial yellow pattern combined with a more important morphological character, the presence/absence of a continuous lateral yellow vitta on scutum, may suggest a clade within *Toxomerus* with phylogenetic importance. Following this argument, Mengual et al. (2011, see also Mengual 2008) recovered a clade with “dark abdomen” species of *Toxomerus* with the lateral yellow scutal vitta interrupted or reduced, which included *Toxomerus anthrax*, *Toxomerus dispar* (Fabricius, 1794) and *Toxomerus flaviplurus* (Hall, 1927).

The two new species, *Toxomerus hauseri* sp. n. and *Toxomerus picudus* sp. n., are very distinct from the other included species. Both have a continuous lateral yellow vitta from postpronotum to scutellum; scutum without a pollinose pattern; eye with triangular emargination large, approximately the half of eye width in lateral view; face yellow with two sublateral black vittae; and abdomen strongly concave, shiny black with lateral margins yellow from tergum 2 to tegum 5. Moreover, *Toxomerus picudus* has a unique morphological character among *Toxomerus* species, a dorsal knob on occiput posterior to ocellar triangle.

## Materials and methods

### Taxonomic revision

Differential diagnoses, synonymies, and distributions are given for all species included in the study. New species are described in full, with terminology following Thompson (1999). Synonymies in full, citations and other references are given in Appendix I. An asterisk (*) in the distribution statement means records from the literature or from *Systema Dipterorum* (Thompson 2010). The acronyms used for collections follow the standard of the *Systema Dipterorum* (Thompson 2010), and their equivalents are listed below:

AMNH American Museum of Natural History, New York, USA.

ANSP Academy of Natural Sciences, Philadelphia, USA.

BMNH The Natural History Museum [formerly the British Museum (Natural History)], London, Great Britain.

CNC Canadian National Collection, Ottawa, Canada.

CSCA California State Collection of Arthropods, Sacramento, USA.

IRSNB Institut Royal des Sciences Naturelles de Belgique, Brussels, Belgium.

MCZ Museum of Comparative Zoology, Cambridge, USA.

MRSN Museo Regionale di Scienze Naturali, Torino, Italy.

MTD Museum für Tierkunde, Dresden, Germany.

MUSM Museo de Historia Natural, Universidad Nacional Mayor de San Marcos, Lima, Peru.

MZSP Museu de Zoologia da Universidade de São Paulo, Sao Paulo, Brazil.

NHRS Naturhistoriska Riksmuseet, Stockholm, Sweden.

NMW Naturhistorisches Museum Wien, Vienna, Austria.

OSU Ohio State University, Columbus, USA.

OUMNH University Museum of Natural History, Oxford, Great Britain.

SMF Forschungsinstitut und Naturmuseum Senckenberg, Frankfurt, Germany.

RMNH Nederlands Centrum voor Biodiversiteit Naturalis [formerly the Nationaal Natuurhistorisch Museum Naturalis], Leiden, The Netherlands.

ZMHB Museum für Naturkunde der Humboldt-Universität, Berlin, Germany.

ZMUCZoological Museum, University of Copenhagen, Copenhagen, Denmark.

In the description of type labels, the contents of each label is enclosed within quotation marks (“ ”) and the individual lines of data are separated by a forward slash ( / ). Complete data for the studied specimens are given in Appendix I. In the material examined section, the use of ellipses follows Standard English practice and merely indicates that the missing information is the same as that in the preceding record. Google Earth was used to find the type locality coordinates of *Toxomerus picudus* sp. n.

All measurements are in millimeters and were taken using a reticule in a Wild M5A microscope. Illustrations of male genitalia were drawn using a camera lucida mounted on an Olympus BX51 compound microscope. Manual drawings were redrawn as a vector image using Adobe Illustrator (version CS3). Photographs were composed using the software CombineZP based on images of pinned specimens taken with a Canon EOS40D mounted on a Microptics Camlift and the help of Adobe Lightroom (version 3.3).

In the identification key, I included four species whose typical form has yellow markings in the center of the abdomen, i.e. *Toxomerus hieroglyphicus* (Schiner, 1868), *Toxomerus paragrammus* (Schiner, 1868), *Toxomerus incaicus* Sack, 1941 and *Toxomerus dispar*. The reason of this inclusion is the occurrence of dark forms due to the high variability of the abdominal pattern. The species *Toxomerus* sp. 1 (*CR-11*), *Toxomerus* sp. 2 (*75-5*) and *Toxomerus* sp. 3 (*CR-B*) are new species to science discovered by F. C. Thompson (USNM, Smithsonian Institution). These codes by F. C. Thompson are placeholders for undescribed species, and are widely used among people working on Syrphidae. Here, I used them for taxa that will be formally described in the future by F. C. Thompson. These codes are not species names and are not valid descriptions according to the code.

### Identification key for the Toxomerus species with dark abdomens

**Table:** 

1	Abdomen with marking pattern; black in background with medial yellow markings either maculae, vittae or fasciae, or abdomen with terga 3-6 yellow (see Figs 1, 2, 4)	other species of *Toxomerus*
–	Abdomen black, sometimes reddish posteriorly, without clear medial/central yellow markings (Figs 3, 10, 12). Abdominal terga with or without yellow lateral margins, continuously yellow from tergum 2 to tergum 5 or interrupted yellow lateral margins (Figs 5, 17, 19)	2
2	Proepimeron black, yellow macula above procoxa absent (Figs 14, 15); scutum variable	3
–	Proepimeron with yellow macula (Fig. 13); scutum with medial white pollinose vitta broadening on posterior margin forming large pollinose macula anterior to scutellum	sp. 1
3	Scutum all dark [usually only postpronotum yellow] or with lateral yellow vitta interrupted, either between postpronotum and transverse suture or ending at transverse suture (Figs 14, 24, 25)	10
–	Scutum with lateral yellow vitta continuous and extending from postpronotum to scutellum (Figs 16, 18)	4
4	Scutellum entirely yellow, black pilose (Figs 26, 27)	9
–	Scutellum black with lateral and posterior margins yellow (Figs 17, 19), sometimes yellow margins not well differentiated from central disc covered by bronze pollinosity, black and/or yellow pilose	5
5	Wing bare basally; costal cell bare on basal half or more, cells CuP, BM and R partly bare (Fig. 6)	7
–	Wing entirely microtrichose, slightly brownish (Borges and Couri 2009: 14, Fig. 13)	6
6	Scutum and scutellum black pilose. Male genitalia with postanal process very reduced. Females with face yellow medially, dark lateroventrally	sp. 2
–	Scutum and scutellum entirely yellow pilose. Male genitalia with postanal process long, more than half as long as surstylus (Borges and Couri 2009: 21, Fig. 47). Females with yellow face with medial broad black vitta	*Toxomerus flaviplurus* (Hall)
7	Face yellow with two sublateral black vittae (Figs 20, 21); profemur yellow, mesofemur partly yellow; cell BM bare on basal third and on anterior and posterior margins. Eye with triangular emargination large: approximately the half of eye width in lateral view (Fig. 23)	8
–	Face yellow; pro- and mesofemora mostly black, yellow very basally and apically; cell BM microtrichose on apical 1/4 with microtrichia extending more basally on posterior margin. Eye with triangular emargination small: at most a third of eye width in lateral view (Fig. 22)	*Toxomerus hieroglyphicus* (Schiner) [dark form]
8	Costal cell entirely bare, at most few microtrichia apically; metafemur black apically, yellow on basal third; occiput with dorsal knob posterior to ocellar triangle pointing posteriorly (Fig. 23) (male unknown)	*Toxomerus picudus* Mengual sp. n.
–	Costal cell bare basally, microtrichose on apical third; metafemur entirely black, at most the apical edge yellow; occiput rounded posteriorly on dorsal section, without any protuberance (Fig. 19) (male unknown)	*Toxomerus hauseri* Mengual sp. n.
9	Wing entirely microtrichose; scutum and notopleuron yellow and black pilose	*Toxomerus paragrammus* (Schiner) [dark form]
–	Wing microtrichose with small bare areas: cells CuP, BM and R bare on anterior margin; scutum and notopleuron entirely yellow pilose	*Toxomerus incaicus* Sack [dark form]
10	Wing bare basally; cell BM bare on anterobasal third or more, cell R1 bare anterior to RS furcation (Fig. 6)	13
–	Wing almost entirely microtrichose, in some cases only a bare line following vein M on anterior margin of cell BM and on posterior margin of cell R	11
11	Katepisternal yellow macula well developed (Figs 13, 15). Pro- and mesotibiae yellow with subapical or medial brown to black ring, less evident in the protibia; wing hyaline, extensively microtrichose with small bare area on both sides of vein M on the basal portion of cell bm, and costal cell bare very basally; in males, antennal bases yellow dorsally and laterally, medial black facial vitta not surrounding antennal bases; in females, abdomen shiny or matte black with no pollen pattern	12
–	Katepisternal yellow macula absent or greatly reduced (Fig. 14). Pro- and mesotibiae bright yellow; wing slightly infuscated, brownish, entirely microtrichose; in males, medial black facial vitta surrounding antennal bases forming narrow dark area between antennal bases; in females, abdomen with black pollen pattern forming at least a black pollinose fascia on tergum 2 (Fig. 10)	*Toxomerus flaviplurus* (Hall)
12	Notopleuron yellow; supra-alar area dark and post-alar callus yellow (http://www.eol.org/data_objects/11884429); scutellum black with broad yellow vitta on lateral and apical margins. Male genitalia: postanal process narrower, as long as or a bit longer than basal width (Fig. 28). Female unknown	*Toxomerus circumcinctus* (Enderlein)
–	Notopleuron black, at most with triangular small yellow macula anteriorly, with a submedial position, not on the most lateral margin (Fig. 15); supra-alar area dark and post-alar callus dark brown; scutellum black basally and laterally, yellow only apically. Male genitalia: postanal process more triangular, broader at the base than long (Fig. 29)	*Toxomerus anthrax* (Schiner)
13	Metacoxa brown to black; metafemur black, at most basal and apical apices yellowish; abdomen entirely black or with yellow vitta on lateral margins, continuous or briefly interrupted at posterior margins of each tergum or abdomen with terga 3–6 yellowish-red	14
–	Metacoxa yellow; metafemur black, yellow on basal 1/4–1/3; abdomen black with terga 2, 3, 4 and 5 with triangular yellow macula on anterior 1/2–2/3 of lateral margins (Borges and Couri 2009: 217, Figs 22, 23)	*Toxomerus basalis* (Walker) [dark form]
14	Pro- and mesofemora dark brown to black, at least on basal half, with apical apex yellow; katepisternal yellow macula broad, broader than anepisternal yellow vitta and normally wider than mesofemur (Figs 13, 24, 25); scutum with a different pattern	15
–	Pro- and mesofemora entirely yellow (male unknown); katepisternal yellow macula reduced, as broad as anepisternal yellow vitta, as wide as or narrower than mesofemur (see Fig. 14); scutum with three broad blue-steel pollinose vittae divided by two submedial brown-bronze pollinose vittae (http://www.eol.org/data_objects/11884446)	*Toxomerus funestus* (Doesburg)
	*Note:* Some specimens of *Toxomerus dispar* may have pro- and mesofemora entirely yellow but they also have katepisternal yellow macula broad and usually abdomen mainly yellow or with evident yellow markings (Figs 1, 2, 4).
15	Postpronotum bright yellow (Fig. 24); notopleuron black or yellow. Postanal process of male genitalia variable	16
	Postpronotum dark brown to black (Fig. 25); notopleuron black. Male genitalia with postanal process short, little distinct, much less than half as long as surstylus (Borges and Couri 2009: 22, Fig. 56)	*Toxomerus laenas* (Walker)
16	Male face entirely yellow; female face with medial black vitta. Notopleuron entirely black. Female abdomen black, shiny or matte, without clear pollinose pattern, sometimes with small yellow areas along lateral margins or lateral margins entirely yellow	17
–	Male and female face with medial black vitta. Notopleuron partly yellow, usually with triangular small yellow macula anteriorly (Fig. 24). Female abdomen usually shiny black with black lateral margins, or terga 4 and 5 with lateral yellow margins; tergum 2 with submedial black pollinose fascia and terga 3 and 4 with four black pollinose vittate maculae (Fig. 9)	*Toxomerus nitidus* (Schiner)
	*Note:* Some specimens of *Toxomerus dispar* may have notopleuron entirely yellow but they also have abdomen mainly yellow or with evident yellow markings (Figs 1, 2, 4).
17	Abdomen partially black on lateral margins: male usually with terga 1 and 2 black, and following terga dark reddish-brown; female usually entirely black (Figs 3, 5). Pro- and mesotibiae yellow. Postanal process of male genitalia long, more than half as long as surstylus (Fig. 8). Females with metaepisternum partly or entirely yellow	*Toxomerus dispar* (Fabricius) [dark form]
–	Abdomen uniformly black with continuous yellow on lateral margins, from tergum 2 to apex. Pro- and mesotibiae yellow, usually with medial dark ring of variable length. Male genitalia with postanal process short, much less than half as long as surstylus. Females with metaepisternum entirely black	sp. 3

## Species accounts

***Toxomerus* sp. 1 (*CR–11*)**

Figure 13

**Differential diagnosis.** Species with face yellow in male and female. Scutum black pilose with medial white pollinose vitta broadening on posterior margin forming a large pollinose macula anterior to scutellum, with a continuous lateral yellow vitta that sometimes looks like interrupted after transverse suture, scutellum entirely black and proepimeron with a yellow macula (Fig. 13). *Toxomerus* sp. 1 has the abdomen metallic blue with black pollinose vittae and fasciae.

**Length** (4): body, 6.8–7.1 (6.9) mm; wing, 5.3–5.8 (5.6) mm.

**Distribution.** Cocos Island (Costa Rica).

**Material examined.** 2♂ 2♀.

***Toxomerus* sp. 2 (75–5)**

**Differential diagnosis.** Species with yellow face medially, brown to black laterally, white pollinose laterally; scutum bronze pollinose with lateral yellow vitta. Scutellum black with lateral and posterior margins yellow, sometimes yellow margins not well differentiated from central disc covered by bronze pollinosity, black and/or yellow pilose. Wing entirely microtrichose. Male abdomen is shiny black with a central black pollinose macula on terga 2 to 5 and small yellow macula on each anterobasal half of terga 2–4; tergum 5 yellow on lateral margins. Female abdomen similar but medial black pollinose macula extended laterally forming a fascia on terga 2 to 5, and lateral yellow macula extending and broadening towards medial line.

**Length** (5): body, 6.4–7.1 (6.8) mm; wing, 6.1–6.2 (6.2) mm.

**Distribution.** Costa Rica.

**Material examined.** 8♂ 7♀.

***Toxomerus* sp. 3(*CR–B*)**

**Differential diagnosis.** Species with yellow face in male, females with a medial broad black vitta continuing along frons and ending at vertex. Scutum black, bronze pollinose wit a medial whitish pollinose vitta and two submedial brown vittae; scutum black laterally except postpronotum yellow, at least on posterior half. Scutellum black, sometimes with apical margin yellow, pale pilose. Wing partly bare, costal cell bare only very basally, cell BM bare on basal half and anterior margin, cell CuP bare basally, cells R and R1 bare before bifurcation RS. Abdomen black, in some specimens becoming reddish at terga 4 and 5, with lateral margins yellow, although this character is not present in all the studied specimens.

Species very similar to *Toxomerus dispar*, but *Toxomerus* sp. 3 has pro- and mesotibiae yellow usually with medial dark ring of variable **length**, the postanal process of the male genitalia is short, much less than half as long as surstylus, and female has metaepisternum entirely black.

**Length** (5): body, 6.3–7.1 (6.7) mm; wing, 5.0–5.9 (5.5) mm.

**Distribution.** Costa Rica.

**Material examined.** 11♀ 8♂.

### Toxomerus anthrax

(Schiner)

http://species-id.net/wiki/Toxomerus_anthrax

Figures 15 29

Mesogramma anthrax Schiner 1868: 350. Type Locality: South America [ST 3♂, 3♀, NMW].
Mesogramma vitrescens Hull 1930: 142. Type locality: Colombia, Magdalena, Aracataca [HT ♂, ANSP].
Mesogramma anthrax var. *flammaria*Hull 1943: 27. Type locality: Honduras, Puerto Castilla [HT ♂, unknown].

#### Differential diagnosis.

Male and female with medial black facial vitta, but in males does not continue laterad or dorsad antennal bases. Scutum black laterally except postpronotum yellow and notopleuron sometimes with a small yellow vitta sublaterally, not on the most lateral margin. Wing extensivey microtrichose with small bare area on both sides of vein M on the basal portion of cell bm, and costal cell bare very basally or along vein SC (posterior margin). Abdomen black, terga 3–5 sometimes dark brown to yellowish-orange, with or without yellow lateral margins; shiny, without pollinose markings.

*Toxomerus anthrax* is similar to *Toxomerus circumcinctus* but the last has the notopleuron entirely yellow and postalar callus yellowish and the scutellum black with a well-defined broad yellow margin. Male genitalia are different.

#### Length

(5): body, 6.0–7.2 (6.6) mm; wing, 4.8–5.5 (5.2) mm.

#### Distribution.

Costa Rica, Venezuela, Colombia, Ecuador, Guyana*, Peru*.

#### Material examined.

2♂ syntypes, 28♂ 17♀.

**Figures 1–15.**
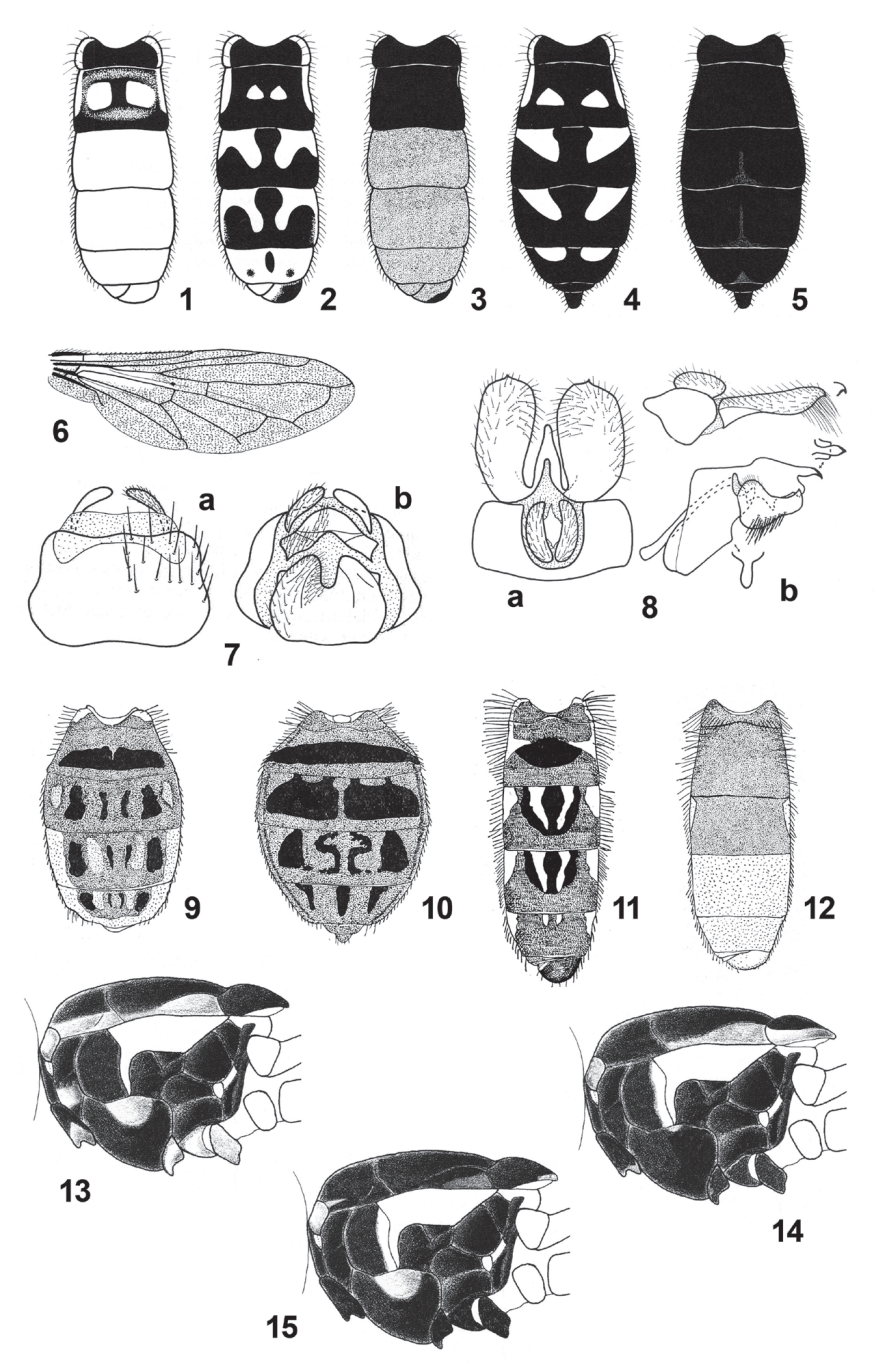
1**–**8 *Toxomerus dispar*: **1** Abdomen of pale male, dorsal **2** Abdomen of dark male, dorsal **3** Abdomen of very dark male, dorsal **4** Abdomen of dark female, dorsal **5** Abdomen of very dark male, dorsal **6** wing **7** Female genitalia: a dorsal, b ventral **8** Male genitalia, 9th tergum and associated structures: a dorsal b lateral **9** *Toxomerus nitidus*, abdomen of female, dorsal **10** *Toxomerus flaviplurus*, abdomen of female, dorsal **11** *Toxomerus hieroglyphicus*, abdomen of male, dorsal **12** *Toxomerus laenas*, abdomen of male, dorsal **13** *Toxomerus* sp. 1, thorax, lateral **14** *Toxomerus flaviplurus*, thorax, lateral **15** *Toxomerus anthrax*, thorax, lateral (Figs 1, 2, 4, 6, 7 and 8 from Thompson 1981; Figs 9**–**15 from Hull 1943).

**Figures 16–29.**
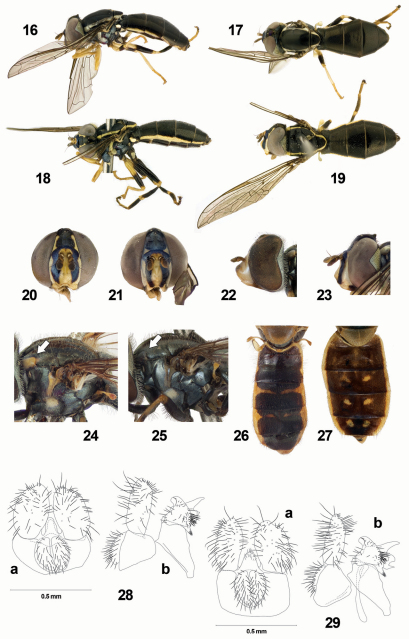
**16** *Toxomerus picudus*, lateral **17** *Toxomerus picudus*, dorsal **18** *Toxomerus hauseri*, lateral **19** *Toxomerus hauseri*, dorsal **20** *Toxomerus hauseri*, head, frontal **21** *Toxomerus picudus*, head, frontal **22 ***Toxomerus hieroglyphicus*, head, lateral **23** *Toxomerus picudus*, head, lateral **24** *Toxomerus nitidus*, thorax, lateral **25** *Toxomerus laenas*, thorax, lateral **26** *Toxomerus paragrammus*, abdomen, dorsal **27** *Toxomerus incaicus*, abdomen, dorsal **28** *Toxomerus circumcinctus*, male genitalia, 9th tergum and associated structures: a dorsal b lateral **29** *Toxomerus anthrax*, male genitalia, 9th tergum and associated structures: a dorsal b lateral.

### Toxomerus basalis

(Walker)

http://species-id.net/wiki/Toxomerus_basalis

Syrphus basalis Walker 1836: 345. Type Locality: Brazil, St. Paul Island [HT ♂, BMNH].
Syrphus portius Walker 1852: 239. Type Locality: Brazil [T ♂, BMNH].
Mesogramma rhea Hull 1949: 228. Type Locality: Brazil [ST ♂, AMNH].
Mesogramma harlequina Hull 1951: 69. Type Locality: Brazil, São Paulo [HT ♀, MZSP].

#### Differential diagnosis.

Species with yellow face. Scutum black, bronze pollinose with a medial bluish pollinose vitta, yellow pilose; postpronotum yellow and notopleuron black with a small yellow macula posteriorly. Metacoxa yellow and metafemur black, yellow on basal 1/4–1/3. Abdomen shiny black with a medial black pollinose vitta on terga 2 to 5; terga 2, 3, 4 and 5 with triangular yellow macula on anterior 1/2–2/3 of lateral margins (see Borges and Couri 2009: 17, Figs 22 and 23). Diffuse, dark yellow markings might be observed in some pale forms in the center of terga 3 to 5. *Toxomerus basalis* has the wing partially bare, with costal cell bare on basal half, cell R1 bare basal to bifurcation RS, cell R bare basal to bifurcation RS and vein bm-cu, cell BM bare on anterior margin and on basal half of posterior margin, and cell CuP bare on anterior margin.

#### Length

(4): body, 6.0–6.5 (6.3) mm; wing, 5.0–5.2 (5.1) mm.

#### Distribution.

Southeastern Brazil.

#### Material examined.

3♂ 1♀.

#### Remarks.

The illustration of *Mesogramma portia* in Hull (1943: 31, Fig. 17) has two submedial short yellow vittae on terga 3, 4 and 5, forming a U-shaped macula on tergum 5. These submedial yellow vittae are at both sides of the medial black pollinose vitta. This illustration refers to a paler form of *Toxomerus basalis*.

### Toxomerus circumcinctus

(Enderlein) stat. n.

http://species-id.net/wiki/Toxomerus_circumcinctus

http://eol.org/pages/753233/overview

Figure 28

Mesogramma circumcincta Enderlein 1938: 232. Type Locality: Peru, Lima [HT ♂, ZMHB].
Toxomerus circumcinctus as synonym of *Toxomerus marginatus*: Thompson et al. 1976: 52 (cat.).

#### Differential diagnosis.

Species with yellow face and a medial broad black vitta, which ends ventrad to antennal bases. Scutum black, yellow pilose, with postpronotum and notopleuron entirely yellow; supra-alar area looks black, although it might have a thin yellow vitta connecting to the yellow postalar callus. Scutellum black with lateral and apical margins yellow. Pleuron black except katepisternum with a dorsal broad yellow macula and posterior anepisternum yellow on posterior third. Halter and calypter yellow. Femora dark brown with yellow apical tips, pro- and mesotibia yellow, metatibia brown with basal and apical extremes yellow, and tarsi brown. Abdomen dark brown, becoming yellowish brown on terga 4 and 5, with continuous yellow lateral margins from tergum 1 to tergum 5.

*Toxomerus circumcinctus* is similar to *Toxomerus anthrax* but the last has the notopleuron black and postalar callus dark brown, and the scutellum black with apical margin yellow (Fig. 15). Male genitalia are different (see Figs 28, 29).

#### Length.

body, 6.0 mm; wing, 5.1 mm.

#### Distribution.

Species only known from the holotype, collected in Peru.

#### Material examined.

♂ holotype.

#### Remarks.

After the original publication (Enderlein 1938), this species has been only cited in two works. Fluke (1956) listed *Toxomerus circumcinctus* in his Neotropical catalogue as *Mesograpta circumcinctum*, and Thompson et al. (1976) synonymized it with *Toxomerus marginatus* (Say, 1823). *Toxomerus marginatus* occurs from Canada south to Central America and it was introduced in Hawaii (Thompson 2010) and it has a typical yellow/black abdominal pattern (http://eol.org/pages/750927/overview). After my study of the *circumcinctus* type, I consider them two different valid species based on morphological characters and male genitalia.

### Toxomerus dispar

(Fabricius)

http://species-id.net/wiki/Toxomerus_dispar

Figures 1–8

Syrphus dispar Fabricius 1794: 309. Type locality: «Americae meridionalis» restricted to Virgin Islands, St. Croix (Thompson 1981: 86) [ST ♂ ♀, ZMUC, destroyed, see Thompson 1981: 86].
Syrphus basilaris Wiedemann 1830: 143. Type locality: Brazil [HT ♂, SMF].
Syrphus vicinus Macquart 1846: 264. Type locality: Brazil [HT ♀, OUMNH].
Mesogramma soror Schiner 1868: 350. Type locality: “America” [HT ♂, NMW]; Thompson et al., 1976: 48 (cat.).
Syrphus tridentatus Rondani 1868: 24. Type locality: Argentina, Patagonia [HT ♂, unknown].
Syrphus melanogaster Thomson 1869: 495. Type locality: Brazil, Rio de Janeiro [HT ♀, NHRS].
Mesograpta variabilis Wulp 1883: 6. Type locality: Guadeloupe, Delaunay [HT ♂, IRSNB].
Melanostoma annulifera Bigot 1884b: 84. Type locality: Mexico [HT ♀, OUMNH].
Mesograpta trilobata Bigot 1884b: 109. Type locality: Mexico [HT ♂, OUMNH].
Orthonevra annulifera Bigot 1884a: 556. Type locality: Brazil [HT ♀, OUMNH].
Paragus ruficaudatus Bigot 1884a: 541. Type locality: Brazil [HT ♂, BMNH].
Mesogramma –? (*bidentata*) Williston 1891: 25. Type locality: Mexico, Acaguizotla, Chilpancingo, Tepetlapa, Medellin near Vera Cruz, Teapa in Tabasco, Orizaba [ST ♂ ♀, unknown].
Mesogramma bidentatum Giglio-Tos 1893: 49. Type locality: Mexico, Acaguizotla, Chilpancingo, Tetetlapa, Medellin presso Vera Cruz, Orizaba, Tampico [ST 6♂ 3♀, MRSN].
Mesogramma imperialis Curran 1926: 103. Type locality: Jamaica, Blue Castle [HT ♀, BMNH].
Mesogramma lutzi Curran 1930: 7. Type locality: Panama, Canal Zone, Grijoles [HT ♀, AMNH].
Mesogramma basilaris var. *flavocuneus*Hull 1940: 433. Type locality: Honduras, Salada River, near Ceiba [HT ♀, CNC].
Mesogramma triangulata Hull 1942a: 104. Type locality: Paraguay, Villarica [HT ♀, AMNH].
Mesogramma basilaris var. *bifida*Hull 1943: 26. Type locality: Ecuador, Baños [HT ♂, CNC].
Mesogramma basilaris var. *neotropica*Hull 1943: 36.
Mesogramma lutzi var. *fasciata*Hull 1948: 8. Type locality: Venezuela. San Esteban [HT ♀, USNM].

#### Differential diagnosis.

Male has yellow face, females a medial broad black vitta continuing along frons and ending at vertex. Scutum black, bronze pollinose wit a medial whitish pollinose vitta and two submedial brown vittae; scutum black laterally except postpronotum yellow, at least on posterior half. Scutellum black, sometimes with apical margin yellow, pale pilose. Wing partly bare basally. *Toxomerus dispar* has the abdominal pattern very variable, from almost entirely yellow to entirely black (see Figs 1–5), and this is the main reason to have such a high number of synonyms.

Species very similar to *Toxomerus* sp. 3, but *Toxomerus dispar* has pro- and mesotibiae yellow, postanal process of male genitalia long, more than half as long as surstylus and female has metaepisternum partly or entirely yellow. The present key only works for dark forms of this species.

#### Length

(5): body, 6.4–7.1 (6.7) mm; wing, 5.1–5.4 (5.3) mm.

#### Distribution.

Widespread in the New World, from USA to Argentina.

#### Material examined.

More than 100 specimens from Mexico, Honduras, El Salvador, Guatemala, Costa Rica, Panama, Ecuador, Venezuela and Brazil.

### Toxomerus flaviplurus

(Hall)

http://species-id.net/wiki/Toxomerus_flaviplurus

Figures 10, 14

Mesogramma flaviplurus Hall 1927: 239. Type Locality: Guatemala, Puerto Barrios [HT ♀, OSU].

#### Differential diagnosis.

Male with yellow face with a medial broad dark vitta surrounding antennal bases forming narrow dark area between antennal bases and dorsad to antennal bases, black ventrolaterally, yellow pilose, white pollinose laterally. Female face and frons yellow with medial black vitta joining medial black frons vitta until the vertex, surrounding laterally the antennal bases. Scutum black, greenish-brown pollinose with dorsomedial broad bluish pollinose vitta and two submedial bronze pollinose vittae, entirely yellow pilose; postpronotum yellowish-brown, slightly lighter than scutum, notopleuron black; supra-alar area and postalar callus yellowish; scutellum black with broad yellow vitta on lateral and apical margins, pale pilose. Pleuron mostly black, except posterior anepisternum black on posterior third, pale pilose; katepisternum with dorsal yellow macula reduced. Wing membrane light brown, entirely microtrichose. Male abdomen shiny black, pale pilose, with tergum 8 as long or longer than tergum 5; male genitalia with long postanal process (Borges and Couri 2009: 21, Fig. 47). Female abdomen a bit more oval, shiny black with a black pollinose pattern (Fig. 10)

#### Length

(5): body, 7.2–7.7 (7.4) mm; wing, 6.6–6.9 (6.8) mm.

#### Distribution.

Guatemala, Costa Rica, Brazil, Panama, Trinidad*.

#### Material examined.

2♂ paratypes, 50♂ 37♀.

#### Remarks.

*Toxomerus flaviplurus* can have yellow markings in the abdomen, with yellow fasciate vittae on terga 2 to 4 and submedial yellow vittae on terga 3 to 5 (see Borges and Couri 2009: 17, Fig. 28). The species key works for the dark form of this species. Some dark specimens of *Toxomerus flaviplurus* can have an almost continuous lateral yellow vitta on the scutum. For this reason, *Toxomerus flaviplurus* appears in two different couplets in the key.

Reemer (2010) synonymised *flaviplurus* under *Toxomerus costalis* (Wiedemann) based on the overall similarity of these species after studying photographs of the paratypes of *Toxomerus flaviplurus* and the holotype of *Toxomerus costalis*. Reemer (2010: 185, Figs 94, 95) included photographs of new *costalis* material from Surinam and male and female look similar to pale forms of *flaviplurus*. The holotype of *Toxomerus costalis* has glued an abdomen of a *Eupeodes* species; the head is also glued but it is the original. After the study of the paratypes of *flaviplurus* and the holotype of *costalis*, I found only a minor difference that is within the variability range of this species: the holotype of *costalis* has the scutellum with a broad yellow vitta on lateral and apical margins. Based on my limited material of *Toxomerus costalis* and the fact that the holotype lacks the abdomen, I have no morphological characters to disagree with Reemer, but I have molecular evidences to not accept this synonym at this moment. Mengual et al. (2011, but see Mengual 2008) inferred the phylogenetic relationships among the genera *Toxomerus* and *Ocyptamus* Macquart, 1834 and their results placed a specimen of *Toxomerus costalis* from Surinam (identified by M. Reemer) distantly related to a specimen of *Toxomerus flaviplurus* from Venezuela. The study of more material and a broader sample of specimens for DNA studies are required to better understand these taxa.

### Toxomerus funestus

(Doesburg)

http://species-id.net/wiki/Toxomerus_funestus

http://www.eol.org/pages/753247

Mesograpta funesta Doesburg 1966: 65. Type locality: Surinam, Zanderij [HT ♀, RMNH].

#### Differential diagnosis.

The female holotype has yellow face with a medial broad black vitta continuing until the vertex, surrounding antennal bases. Scutum black with three broad blue-steel pollinose vittae divided by two submedial brown-bronze pollinose vittae. Pro- and mesofemora entirely yellow, katepisternal yellow macula reduced, as broad as anepisternal yellow vitta, as wide as or narrower than mesofemur. Wing partly bare basally. Abdomen entirely black with terga 2 to 5 with a medial black pollinose vitta and two submedial triangular macula of black pollen.

*Toxomerus funestus* is a very distinct species with a unique abdominal and scutal patterns, and pro- and mesolegs yellows except coxae, trochanters and femora basally brown.

#### Length:

body, 7.2 mm; wing, 5.4 mm.

#### Distribution.

Surinam, Brazil.

#### Material examined.

♀ holotype.

### Toxomerus hauseri

Mengual sp. n.

urn:lsid:zoobank.org:act:1A51A3E2-FB67-4601-86A2-BCE8B11CB7AE

http://species-id.net/wiki/Toxomerus_hauseri

Figures 18, 19, 20

#### Description.

FEMALE. *Head*: Face with distinct low facial tubercle, which ends at oral margin, yellow with two submedial black vittae that end before the oral margin, brownish lateroventrally, scarcely yellow pilose; gena brown to black; lunule yellow, yellow also between antennal bases; frons yellow laterally with broad medial black vitta that surrounds antennal bases and continues with the two submedial facial vittae, yellow pilose; vertical triangle shiny black, black pilose; antennae on small produced tubercle, antenna orangish, basoflagellomere brown, orange basoventrally; arista brown, bare (Fig. 20); eye bare, lateral triangular eye emargination large, approximately the half of eye width in lateral view; occiput black, grey pollinose, yellow pilose on ventral 2/3 and black pilose on dorsal 1/3.

*Thorax*: Scutum shiny with a continuous lateral yellow vitta, yellow pilose laterally and anteriorly, black pilose posteriorly; postpronotum yellow, bare; notopleuron yellow with a black vitta on the lateral side narrowing the lateral yellow scutal vitta; supra-alar area and postalar callus yellow; scutellum black with well-defined yellow margin apically and laterally, black pilose with a row of bristle-like black pile in the posterior margin, subscutellar fringe absent (Figs 18, 19). Pleuron mostly black, except posterior anepisternum yellow on posterior third and katepisternum with dorsal broad yellow macula; metasternum bare; calypter yellow; plumula yellow; halter bright yellow; posterior spiracular fringes yellow. *Wing*: Wing membrane hyaline, stigma brown; extensively microtrichose, except costal cell bare on basal 2/3, cells R1 and R bare basal to furcation of RS, cell BM bare basally and on anterior margin. Alula microtrichose. *Legs*: Proleg yellow except coxa brown and femur orangish on apical half, yellow pilose; mesoleg yellow except coxa black, mesofemur black on apical half with apical tip yellow, yellow and black pilose; metaleg black except femur yellow on apical tip, tibia yellow on basal tip and on apical 1/6–1/5 (Figs 18, 19).

*Abdomen*: Slightly oval, distinctively convex, unmargined. Dorsum shiny black, black pilose; terga 2, 3, 4 and 5 with lateral margins yellow forming a continuous lateral yellow vitta, barely interrupted at the posterior margin of tergum 4. Sterna shiny black; sterna 1 and 2 with brownish-yellow fascia on posterior margin (Figs 18, 19).

#### Differential diagnosis.

Species with yellow face with two sublateral black vittae, and eye with triangular emargination large, approximately the half of eye width in lateral view. Profemur yellow; cell BM bare on basal third and on anterior and posterior margins. Scutum shiny black with lateral yellow vitta and abdomen shiny black, convex, with lateral margins entirely yellow. Very similar to *Toxomerus picudus* sp. n. but differs by having costal cell microtrichose on apical third, metafemur entirely black with at most the apical edge yellow, and occiput rounded posteriorly on dorsal section, without any protuberance.

#### Length:

body, 6.6 mm; wing, 5.9 mm.

#### Distribution.

Species only known from the holotype, collected in Peru.

#### Etymology.

This species is named after Martin Hauser in recognition of his work on Diptera and his help during my study of flower flies.

#### Type locality.

PERU: Pasco Region, Oxapampa Province, Huancabamba District, Yanachaga-Chemillén N. P., Canon of Huancabamba, Biological Station Huampal, 1050 m, 10°11'08.95"S, 75°34'27.12"W, collected using a Malaise trap, D. Takiya, C. Peña and R. Rakitov leg.

#### Type specimen.

Holotype female, pinned, deposited at Museo de Historia Natural, Universidad Nacional Mayor de San Marcos, Lima, Peru. Original label: “PERU, Pasco 6–9. Oct. 2002 / Yanachaga-Chemillén N. P. / Canon of Huancabamba R. / Biol. Station Huampal, 1050 m / 10,18582°S, 75,57420°W / Malaise trap across river / D. Takiya, C. Peña, R. Rakitov” “HOLOTYPE / *Toxomerus / hauseri / Mengual 2011*” [red, handwritten except first line] (♀, MUSM).

### Toxomerus hieroglyphicus

(Schiner)

http://species-id.net/wiki/Toxomerus_hieroglyphicus

Figures 11 22

Mesogramma hieroglyphica Schiner 1868: 348. Type locality: South America [LT ♂, NMW].

#### Differential diagnosis.

Species with yellow face in male, sometimes with a brownish macula on tubercle. Scutum black, bronze pollinose medially and a medial bluish pollinose vitta, with a continuous lateral yellow vitta from postpronotum to scutellum, narrowed on notopleuron and supra-alar area. Scutellum black with yellow lateral and apical margins. Wing partially bare basally, costal cell bare on basal half or a bit more. Abdomen with medial yellow markings on terga 3 to 5; tergum 1 with yellow lateral margins, tergum 2 black with a yellow macula on anterolateral half extending narrowly towards the center of the tergum, and with a roundish black pollinose macula in the center; tergum 3 and 4 black with anterolateral yellow maculae and two submedial curved yellow vittae that divides a central black pollinose area; tergum 5 black with anterolateral yellow maculae and two submedial short yellow vittae.

*Toxomerus hieroglyphicus* is close in the key to the two new species, *Toxomerus picudus* and *Toxomerus hauseri*, but they are very different as already noted in the key.

#### Length

(4): body, 5.6–6.2 (5.9) mm; wing, 5.0–5.7 (5.2) mm.

#### Distribution.

Venezuela, Ecuador, Colombia*.

#### Material examined.

♂ lectotype, ♂ paralectotype, 3♂.

#### Remarks.

*Toxomerus hieroglyphicus* has usually yellow markings in the center of the abdomen. Thus, it should not be included in the present key. However, the study of a dark specimen prompted me to tentatively include this species in case darker specimens might appear with completely black abdomen with lateral yellow maculae.

### Toxomerus incaicus

Sack

http://species-id.net/wiki/Toxomerus_incaicus

Figure 27

Toxomerus incaicus Sack 1941: 101. Type locality: Peru, Querobamba, and Bolivia [LT ♂, MTD].

#### Differential diagnosis.

Species with yellow face in both sexes, frontal triangle of male yellow and female frons with a medial broad black vitta. Sucutm black, green-grey pollinose with a lateral broad yellow vitta from postpronotum to scutellum. Scutellum yellow, black pilose. Legs entirely yellow except metatarsi dark brown. Wing mostly microtrichose, bare only on anterior margin of cells R, BM and CuP and cell R1 basally. Abdomen black with lateral margins yellow, tergum 1 with anterior margin yellow, and terga 6 to 9 yellow; terga 2 to 5 with a central dark pollinose macula; and terga 3 to 5 with two submedial, small, round yellow maculae.

#### Length

(2): body, 6.0–6.2 (6.1) mm; wing, 5.7–5.8 (5.7) mm.

#### Distribution.

Peru, Bolivia*.

#### Material examined.

1♂ 1♀ paralectotypes.

#### Remarks.

*Toxomerus incaicus* has usually medial yellow maculae on terga 3 to 5. Again, this species should not appear in the present key. However, I included this species in case darker specimens might appear with completely black abdomen with lateral yellow margins.

### Toxomerus laenas

(Walker)

http://species-id.net/wiki/Toxomerus_laenas

Figures 12 25

Syrphus barbulus Walker 1852: 238. Type locality: Brazil [HT ♀, BMNH].
Syrphus laenas Walker 1852: 241. Type locality: Brazil [HT ♂, BMNH].
Mesogramma nitidiventris Curran 1930: 9. Type locality: Brazil, Espirito Santo, Vitoria [HT ♂, AMNH].
Mesogramma vitrea Hull 1941: 45. Type locality: Brazil, São Paulo, Juquia [HT ♂, CNC].

#### Differential diagnosis.

Species with yellow face in male and female with a medial, very broad, black vitta, gena black. Scutum black, green-gray pollinose with a medial white pollinose vitta, dark laterally. Postpronotum black, sometimes brownish posteriorly. Scutellum black, sometimes with apical margin yellow, pale pilose. Wing bare on anterior margin of cells R, BM and CuP and cell R1 basally. Abdomen shiny black, pale pilose; sometimes becoming reddish-brown apically. Male genitalia with postanal process short, little distinct, much less than half as long as surstylus.

#### Length

(5): body, 6.2–6.8 (6.6) mm; wing, 5.7–6.1 (5.8) mm.

#### Distribution.

Venezuela, Brazil, Paraguay*.

#### Material examined.

♂ holotypeof *nitidiventris*, ***Non-type material*:** 12♂ 9♀.

### Toxomerus nitidus

(Schiner)

http://species-id.net/wiki/Toxomerus_nitidus

Figure 24

Mesogramma nitida Schiner 1868: 349. Type locality: South America [Venezuela] [ST ♂, NMW].
Mesogramma ovata Hull 1942b: 19. Type locality: Panama, Yape, Tuirar [HT ♀, MCZ] syn. n.

#### Differential diagnosis.

Species with yellow face and a medial black facial vitta in both sexes, geba black. Scutum black, green-bronze pollinose with a medial bluish-white pollinose vitta, sometimes with two submedial whitish vittae. Postpronotum yellow and notopleuron partly yellow, usually with triangular yellow macula anteriorly narrowing towards transverse suture. Wing partly bare basally, with costal cell entirely microtrichose and brown, darker than the rest of the wing except stigma. Male abdomen usually bicolor, with terga 1 and 2 black (tergum 1 with yellow anterior corners) and terga 3 to 5 reddish-orange; postanal process of the male gentialia long. Female abdomen usually shiny black with black lateral margins; tergum 2 with submedial black pollinose fascia and terga 3 and 4 with four black pollinose vittate maculae (Fig. 9).

Species close to *Toxomerus dispar* and *Toxomerus* sp. 3 but males of *nitidus* have black facial vitta and notopleuron partly yellow.

#### Length

(5): body, 6.1–7.4 (6.9) mm; wing, 5.5–6.9 (6.1) mm.

#### Distribution.

Guatemala, Costa Rica, Panama, Colombia.

#### Material examined.

2♂ syntypes, ♀ holotype of *ovatus*, ***Non-type material*:** 7♂ 10♀.

#### Remarks.

Males of *Toxomerus nitidus* always have a bicolored abdomen and females may have yellowish markings as noted by Hull (1943) (see Fig. 9), although most of the studied specimens had shiny black abdomens with a black pollinose pattern.

*Toxomerus nitidus* has been cited few times after its original description but only in catalogues (see Appendix I). I had the possibility to study two syntypes of *Toxomerus nitidus* and compared them with males of *Toxomerus ovatus* at USNM. Male genitalia were identical and females of *ovatus* did key out as *nitidus*. Thus, I realized that *Toxomerus nitidus* was only known from male specimens. Therefore, *Toxomerus ovatus* is here considered to be a junior synonym of *Toxomerus nitidus*.

### Toxomerus paragrammus

(Schiner)

http://species-id.net/wiki/Toxomerus_paragrammus

Figure 26

Mesogramma paragramma Schiner 1868: 349. Type locality: South America [Venezuela] [ST ♂, NMW].

#### Differential diagnosis.

Species with face produced forward, yellow, with gena brown. Scutum black, bronze pollinose, with a broad yellow lateral vitta, yellow and black pilose. Scutum yellow, black pilose. Pleuron mostly black except posterior anepisternum yellow on posterior 2/3, katepisternum with a dorsal yellow macula and anepimeron yellow on anterior and dorsomedial sections. Wing hyaline, microtrichose. Abdomen mainly black, with a broad yellow lateral margin in terga 1 to 5; tergum 2 with a medial black pollinose macula; terga 3 and 4 with two subanterior fasciate maculae that can eventually meet in the middle with a central yellow vitta (Fig. 26).

#### Length

(2): body, 6.8–7.0 (6.9) mm; wing, 6.3–6.5 (6.4) mm.

#### Distribution.

Venezuela.

#### Material examined.

2♂ syntypes.

#### Remarks.

*Toxomerus paragrammus* is another species that most of the times will not run through the key because the presence of yellow maculae on the abdomen. I included this species because I think some dark specimens might have black abdomen with yellow lateral margins.

### Toxomerus picudus

Mengual sp. n.

urn:lsid:zoobank.org:act:FCDB7EE3-F121-4192-A463-3752E15CC4CA

http://species-id.net/wiki/Toxomerus_picudus

Figures 16, 17, 21, 23

#### Description.

FEMALE. *Head*: Face with distinct low facial tubercle, more pointed forward than rounded, yellow with two submedial black vittae that reach oral margin, brownish lateroventrally, scarcely yellow pilose; gena brown to black; lunule yellow, yellow also between antennal bases; frons yellow laterally with broad medial black vitta that surrounds antennal bases and continues with the two submedial facial vittae, yellow-golden pilose; vertical triangle shiny black, black pilose; antennae on small produced tubercle, antenna orangish, basoflagellomere dark brown dorsally; arista brown, bare (Fig. 21); eye bare, lateral triangular eye emargination large, approximately the half of eye width in lateral view; occiput with dorsal knob posterior to ocellar triangle, black, grey pollinose, yellow pilose on ventral 2/3 and black pilose on dorsal 1/3 (Fig. 23).

*Thorax*: Scutum shiny, bronze pollinose very anteriorly, with a continuous lateral yellow vitta, yellow pilose; postpronotum yellow, bare; notopleuron yellow with a black vitta on the lateral side narrowing the lateral yellow scutal vitta; supra-alar area and postalar callus yellow; scutellum black with well-defined lateral yellow vitta, slightly narrowed apically, black pilose, subscutellar fringe absent (Figs 16, 17). Pleuron mostly black, except posterior anepisternum yellow on posterior third and katepisternum with dorsal broad yellow macula; metasternum bare; calypter yellow; plumula yellow; halter bright yellow; posterior spiracular fringes yellow. *Wing*: Wing membrane hyaline, stigma brown; extensively microtrichose, except costal cell bare, cell R1 bare beyond RS furcation until the middle of the stigma, cell R bare basal to furcation of RS, cell BM bare on basal fourth and on anterior margin, cell CuP bare on anterior margin. Alula microtrichose. *Legs*: Proleg yellow except coxa black, yellow pilose; mesoleg yellow except coxa black, mesofemur black on apical half with apical tip yellow, mesotarsi orangish; metaleg black except femur yellow on basal third and on apical tip, tibia yellow on basal tip and on apical 1/6-1/5 (Figs 16, 17).

*Abdomen*: Slightly oval, distinctively convex, unmargined. Dorsum shiny black, black pilose; terga 2, 3, 4 and 5 with lateral margins yellow forming a continuous lateral yellow vitta, barely interrupted at the posterior margin of tergum 4. Sterna shiny black; sterna 1 and 2 with brownish-yellow fascia on posterior margin (Figs 16, 17).

#### Differential diagnosis.

Species with yellow face with two sublateral black vittae, and eye with triangular emargination large, approximately the half of eye width in lateral view. Profemur yellow; cell BM bare on basal third and on anterior and posterior margins. Scutum shiny black with lateral yellow vitta and abdomen shiny black, convex, with lateral margins entirely yellow. Very similar to *Toxomerus hauseri* sp. n. but differs by having costal cell entirely bare, at most few microtrichia apically, metafemur black apically, yellow on basal third, and occiput with dorsal knob posterior to ocellar triangle pointing posteriorly. Moreover, the submedial black facial vittae reach oral margin in *Toxomerus picudus* but not in *Toxomerus hauseri*.

#### Length

**:** body, 6.2 mm; wing, 5.1 mm.

#### Distribution.

Ecuador.

#### Etymology.

The specific epithet is derived from the Spanish *picudo* that means having a knob, protuberance. It refers to the dorsal occipital knob that this species has. Species epithet is treated as adjective.

#### Type locality.

ECUADOR: Orellana Province, Aguarico Canton, Tiputini Biodiversity Station, 227 m., 0°38'13.73"S, 76°08'59.62"W, collected using a Sante trap, upper jar, A. Tishechkin leg.

#### Type specimen.

Holotype female, pinned. Original label: “ECUADOR: Orellana Prov, Tiputini / Biodiversity Stan, Canopy 30m, Sante / trap, upper jar, 29.VII-3.VIII.2008. / AT1090, A. Tishechkin” “HOLOTYPE / *Toxomerus / picudus / Mengual 2011*” [red, handwritten except first line] (♀, CSCA).

## Supplementary Material

XML Treatment for Toxomerus anthrax

XML Treatment for Toxomerus basalis

XML Treatment for Toxomerus circumcinctus

XML Treatment for Toxomerus dispar

XML Treatment for Toxomerus flaviplurus

XML Treatment for Toxomerus funestus

XML Treatment for Toxomerus hauseri

XML Treatment for Toxomerus hieroglyphicus

XML Treatment for Toxomerus incaicus

XML Treatment for Toxomerus laenas

XML Treatment for Toxomerus nitidus

XML Treatment for Toxomerus paragrammus

XML Treatment for Toxomerus picudus
